# Diet Quality, Food Insecurity, and Chronic Kidney Disease: Insights From the US National Health and Nutrition Examination Survey

**DOI:** 10.1053/j.jrn.2025.10.004

**Published:** 2025-10-23

**Authors:** Hiba Hamdan, Tae Youn Kim, Laura C. Plantinga, Hilary Seligman, Deidra C. Crews, Delphine S. Tuot

**Affiliations:** * Division of Nephrology, Department of Medicine, University of California Davis Health, Sacramento, California.; † Betty Irene Moore School of Nursing, University of California Davis Health, Sacramento, California.; ‡ Division of Rheumatology at Zuckerberg San Francisco General Hospital, University of California, San Francisco, San Francisco, CA.; § Division of Nephrology at Zuckerberg San Francisco General Hospital, University of California, San Francisco, San Francisco, CA.; ¶ Division of General Internal Medicine at Zuckerberg San Francisco General Hospital, University of California, San Francisco, San Francisco, CA.; ** ARC for Health Equity, University of California, San Francisco, San Francisco, CA.; †† Division of Nephrology, Department of Medicine, Johns Hopkins University School of Medicine; Baltimore, MD.

**Keywords:** diet quality, chronic kidney disease, food insecurity, NHANES

## Abstract

**Objectives:**

Healthy eating patterns may prevent development of chronic kidney disease (CKD). Food insecurity is a known barrier to high diet quality. We examine the association of diet quality with CKD prevalence and food insecurity in a nationally representative population.

**Methods:**

Cross-sectional analysis of data was conducted among eligible adults (n = 5,974) in the 2017–2020 National Health and Nutrition Examination Survey. Multivariable linear and logistic regression models were used to examine the association between diet quality, assessed using the Healthy Eating Index (HEI)-2020, and CKD prevalence and between food insecurity, assessed using a 10-item questionnaire, and diet quality. CKD was defined as an estimated glomerular filtration rate <60 mL/min per 1.73 m^2^ or urine albumin-to-creatinine ratio ≥30 mg/g. Models adjusted for sociodemographic and health characteristics were conducted in the general population, among people with diabetes, and those with hypertension.

**Results:**

Diet quality was not significantly associated with CKD prevalence in the general population (adjusted odds ratio [aOR] per 10-point increase = 0.88; 95% confidence interval (CI): 0.78, 1.01). However, a 10-point higher HEI-2020 score was associated with 24% lower odds of CKD in those with diabetes (aOR = 0.76; 95% CI: 0.61, 0.95) and 20% lower odds in those with hypertension (aOR = 0.80; 95% CI: 0.69, 0.99). In adjusted models, no statistically significant association was observed between diet quality and food insecurity.

**Conclusions:**

Poorer diet quality was associated with a higher prevalence of CKD among individuals with diabetes and/or hypertension. Addressing barriers to healthy eating is essential among individuals at risk for CKD.

## Introduction

The impact OF diet on health is increasingly evident, particularly in its relationship with the incidence and progression of chronic diseases such as obesity, diabetes mellitus (DM), hypertension (HTN), and chronic kidney disease (CKD).^1–4^ Consumption of high-quality diets rich in fruits, vegetables, and whole grains and low in sodium, added sugars, saturated fats, processed foods, and red meats—such as the Dietary Approaches to Stop Hypertension diet, the Mediterranean diet, and plant-based diet—has been associated with improved cardiovascular outcomes, better blood pressure and glycemic control, and weight loss among individuals with HTN, DM, heart disease, and obesity, all of whom are at high risk for CKD.^5–7^

In response to the critical link between diet quality and health, the Food as Medicine initiative was launched by the Office of Disease Prevention and Health Promotion in 2022 to focus on integrating into medical care consistent access to diet and nutrition-related resources to eliminate hunger and reduce the prevalence of chronic disease in the United States.^8^ Food as Medicine has gained momentum in developing solutions to improve diet quality through programs such as medically tailored meals and produce grocery prescriptions, especially for patients with diabetes and heart failure.^9,10^ People with or at risk for kidney disease may stand to gain significant benefits from improved diet quality.

Understanding many factors that shape diet quality in the US is important to guide efforts to improve it for our patients. Americans identify the cost of food as being a primary barrier to healthy dietary intake.^11^ Food insecurity—defined as limited or uncertain access to adequate food—has risen in recent years, affecting 17 million US^12^ households in 2022, up from 13.8 million in 2020. It has been linked to chronic diseases such as obesity, HTN, DM, cardiovascular disease, and CKD.^13–15^ However, because food insecurity was conceptualized as an economic indicator, our measurement tools may better assess access to sufficient quantity, rather than quality, of food. Thus, food insecurity might be a surrogate marker for other barriers to high-quality diets, such as poverty or lower education.

To evaluate how diet quality affects the prevalence of CKD and help understand the effect of food insecurity on diet quality, we examined the relationships between diet quality, food insecurity, and CKD prevalence using recent nationally representative data. We hypothesized that diet quality among individuals at risk for CKD is poor, that poor diet quality is associated with higher prevalence of CKD, and that food insecurity is associated with poor diet quality.

## Methods

We examined data from the 2017–2020 survey periods of the publicly available National Health and Nutrition Examination Survey (NHANES). NHANES is conducted by the National Center for Health Statistics of the Centers for Disease Control and Prevention to provide information on health and nutritional status in representative samples of noninstitutionalized US civilian residents. The survey consists of a standardized in-home interview and a physical examination and blood and urine collection at mobile examination centers. NHANES uses a complex multistage probability sampling design. This study was deemed exempt from institutional review board (IRB) approval because it involved the analysis of publicly available, de-identified data from NHANES.

The 2017–2020 files represent a unique public use data released from NHANES. The coronavirus disease 2019 (COVID-19) pandemic required suspension of data collection in March 2020. As a result, the partially completed NHANES 2019–2020 cycle was not nationally representative. Therefore, the 2019-March 2020 data were combined with the data from the 2017–2018 cycle to create the nationally representative 2017-March 2020 prepandemic dataset. The 2019–2020 primary sampling units were reassigned to the 2015–2018 sample design strata and merged with the 2017–2018 data to generate a dataset suitable for producing nationally representative estimates. A primary sampling unit-level adjustment factor was applied to balance each stratum's contribution to the overall survey sample, which was then incorporated into the participant base weights. Interview and examination weights were derived from these adjusted base weights.^16^

Our population of interest included US adults aged 18 years or older who had two 24-hour dietary recalls in the 2017–2020 NHANES (n = 6,961). Of these, 6,605 had food insecurity data available. We excluded individuals who were pregnant (n = 64), those for whom CKD status could not be ascertained (n = 254), and those with total daily calorie intake less than 500 or more than 5000 kcals to reduce potential misreporting (n = 313), resulting in a final analytic sample of 5,974 participants.

### Measurements and Definitions

Diet quality was assessed using the Healthy Eating Index (HEI)-2020, which is calculated from two 24-hour dietary recalls. An initial dietary recall was conducted at the Mobile Examination Center, and a second dietary recall was completed by phone. Scores were calculated using the simple HEI-2020 scoring algorithm available in the dietary index package for R version 1.0.3.^17,18^

HEI was originally developed in 1995 as a tool to evaluate the extent to which Americans were following dietary recommendations according to the Dietary Guidelines for Americans. In 2005, the structure of the HEI was revised and has been updated three times since then as a collaboration between the US Department of Agriculture, Food and Nutrition Services, Center for Nutrition Policy and Promotion, and Health and Human Services National Cancer Institute.^19^ The HEI has a total score of 100 points, divided into 13 components: total fruits, whole fruits, total vegetables, greens and beans, whole grains, dairy, total protein foods, seafood and plant proteins, fatty acids, refined grains, sodium, added sugars, and saturated fats. All components are scored positively except for refined grains, sodium, added sugars, and saturated fats, which are reverse scored. Maximum scores are five points for total fruits, whole fruits, total vegetables, greens and beans, total protein foods, and seafood and plant proteins and 10 points for the remaining components.^19,20^ HEI scores greater than 80 indicate a “good” diet, scores between 51 and 80 reflect a diet that “needs improvement,” and scores below 51 suggest a “poor” diet.^20^

Food insecurity over the prior 12 months was assessed using a 10-item well-validated questionnaire developed by the US Department of Agriculture. We grouped the food security status into three categories: food secure (0 affirmative responses), marginally food secure (1–2 affirmative responses), and food insecure (3–10 affirmative responses). Only items pertaining to adults in the household were used in this categorization.^21^

Demographic information (age, sex, race/ethnicity, and marital status), socioeconomic status (education and household income), health behaviors (having ever smoked), and health conditions (HTN and DM) were collected during the survey interviews. Income was measured using the poverty income ratio, which compares household income to the federal poverty level. Ratios <1.0 indicate a household income below the poverty level, whereas ratios >1.0 indicate a household income above the poverty level. We divided the poverty income ratio into four categories (<1.3, 1.3–1.85, 1.85–2.85, and >2.85); these cut-points were chosen to align with common thresholds used to qualify for federal nutrition assistance programs.^22,23^ Body mass index was calculated by dividing weight (kg) by height (m) squared, and obesity was defined as body mass index >30 kg/m^2^. Diabetes was identified based on self-report, use of oral diabetes medications or insulin, a measured nonfasting plasma glucose ≥200 mg/dL, or glycosylated hemoglobin (HbA1c) > 6.5%. Hypertension was determined by self-report, use of antihypertensive medications, or measured average systolic blood pressure (SBP) > 130 mmHg or diastolic blood pressure (DBP) > 90 mmHg.

Serum creatinine was measured using the enzymatic creatininase Roche Cobas 6000 method. Urine albumin was quantified through fluorescein immunoassay with the SequoiaTurner Digital Fluorometer (Model 450), and urine creatinine was analyzed using the Enzymatic Roche Cobas 6000 Analyzer, all conducted at the same laboratory.

Estimated glomerular filtration rate (eGFR) was calculated using the 2021 CKD-EPI (Chronic Kidney Disease Epidemiology Collaboration) equation.^24^ Albuminuria was considered present at urinary albumin-to-creatinine ratios ≥30 mg/g. CKD was defined using single values of eGFR <60 mL/min per 1.73 m^2^ or presence of albuminuria, based on the Kidney Disease Improving Global Outcomes Guidelines.^24^

### Statistical Methods

Weighted analyses were conducted to represent the noninstitutionalized US civilian population aged 18 years and older. Complex survey procedures using R survey package version 4.4.2 were used to generate weighted estimates, standard errors (SEs), and *P* values while accounting for the stratification and clustering inherent in the design of NHANES.^25,26^ The recommended 3-year dietary weights for participants with completed day 1 and day 2 recalls were applied. A *P*-value threshold of <0.05 was considered statistically significant.

Participant characteristics, both overall and by DM or HTN diagnosis, were compared using descriptive statistics. For categorical variables, survey-weighted percentages and unweighted counts were reported. For continuous variables, survey-weighted means and medians, along with their corresponding standard errors and interquartile ranges, were reported as appropriate.

Multivariable logistic regression was used to determine the magnitude and direction of the relationship between diet quality and CKD status using unadjusted models with sequential adjustment for demographic and social factors, including education level and poverty income ratio category and chronic diseases, and conditions, including DM, HTN, and obesity. Subgroup analyses by DM and HTN status were performed to determine whether diet quality was differentially related to CKD among individuals with DM and HTN.

Multivariable linear regression models were used to examine absolute differences in HEI-2020 total scores by food insecurity status in the general population, reporting an unadjusted model and sequentially adjusting for the aforementioned demographic, social, and disease status variables. Subgroup analyses by DM and HTN status were conducted to assess whether the association between food security and diet quality varied among individuals with DM and HTN. As all variables had less than 10% missing-ness, participants with missing exposure and outcome data were excluded from the analysis.

## Results

### Weighted Participant Characteristics Overall

The weighted mean age of participants was 49 years (SD = 17.4), and 52% were female. The racial/ethnic distribution was 64% White, 11% Black, and 16% Hispanic. Overall, 64% had a college degree or some college education. The weighted prevalence of DM, HTN, and obesity was 14%, 46%, and 42%, respectively. Among US adults aged 18 years and older during the 2017–2020 period, nearly one-sixth of the US population (16%) were classified as food insecure, and 10% were marginally food secure (Table 1 and Supplemental Table 6).

### Association Between Diet Quality and Prevalence of Chronic Kidney Disease

Diet quality, as measured by the HEI-2020, had a mean value of 50.9 ± 12.2 in the general population, 51.6 ± 12.2 in the adult population with diabetes, and 51.2 ± 11.9 among those with HTN.

In a weighted unadjusted logistic regression model, diet quality was not statistically significantly associated with CKD prevalence (unadjusted odds ratio per 10-point increase = 0.95; 95% confidence interval (CI): 0.87, 1.05). However, after adjusting for sociodemographic variables, education level, poverty income ratio category, and comorbidities, diet quality was not statistically significantly associated with CKD prevalence in the general population (adjusted odds ratio per 10-point increase = 0.88; 95% CI: 0.78, 1.01). A stronger association between diet quality and CKD prevalence was seen among the subpopulations of individuals with DM and HTN. Among individuals with diabetes, in a fully adjusted model, each 10-point higher HEI-2020 score was associated with a 24% reduction in the odds of CKD prevalence (adjusted odds ratio = 0.76; 95% CI: 0.61, 0.95). Among individuals with hypertension, in a fully adjusted model, each 10-point higher HEI-2020 score was associated with 20% lower odds of CKD prevalence (adjusted odds ratio = 0.80; 95% CI: 0.69, 0.99) (Table 2 and Supplemental Tables 1 and 2).

### Association Between Food Insecurity and Diet Quality

HEI-2020 scores were lower among individuals with higher food insecurity (mean = 52.1, 48.2, and 47.5 for food secure, marginally food secure, and food insecure, respectively; *P* < .001). An unadjusted linear regression model revealed that greater food insecurity was statistically significantly associated with lower total HEI scores in the overall population. Individuals experiencing food insecurity (−4.53, 95% CI: −5.73, −3.33) or marginal food security (−3.83, 95% CI: −5.58, −2.07) had significantly lower HEI scores compared to those who were food secure. After adjusting for sociodemographic variables, including education level and poverty income ratio category, the association between food security status and diet quality was no longer statistically significant (−1.68, 95% CI: −3.46, 0.10 for food insecurity and −1.01, 95% CI: −3.18, 1.15 for marginal food security). The association remained statistically nonsignificant after adjustment for chronic conditions in the overall population (Table 3 and Supplemental Table 3).

Similar patterns were observed among individuals at high risk for CKD due to HTN. In this subgroup, food insecurity but not marginal food security was associated with lower HEI scores in unadjusted models, but the associations were not statistically significant after adjustment for sociodemographic variables. Among individuals at high risk for CKD due to DM, food insecurity was not statistically associated with diet quality in both unadjusted and adjusted models (Supplemental Tables 4 and 5).

## Discussion

In this study, using recently collected nationally representative data, we show that diet quality is generally poor in the adult US population and that poorer diet quality is associated with a higher prevalence of CKD, particularly among individuals at heightened risk of kidney disease due to DM and/or HTN. These findings underscore the role of diet quality in CKD prevention and management, highlighting the need for interventions that improve diet quality for individuals with or at risk for CKD. Additionally, we show that despite an association between food insecurity and diet quality in the overall population in an unadjusted model, the association is attenuated after adjustment for education and income levels. These data highlight the complex relationship between food insecurity and diet quality and serve as a call for more studies to understand this relationship in an effort to improve diet quality for individuals facing the most systemic barriers to healthy lifestyles who are also at highest risk for diet-related chronic diseases.^27^

Despite growing recognition of the important role of high-quality diets in the prevention and management of chronic diseases such as DM, obesity, HTN, and CKD, the overall quality of the American diet is suboptimal. In this NHANES population, we found average diet quality to be poor overall and among people with DM and those with HTN. People with food security had better diet quality compared to those with food insecurity. This is somewhat consistent with the findings from Liu et al, who showed that using NHANES data despite some improvement in diet quality between 1999 and 2020 among those with food security, diet quality didn't improve among those experiencing food insecurity over the same time period.^27^ These findings are particularly relevant, as the leading causes of death in the United States are now largely driven by modifiable lifestyle factors, including tobacco use, unhealthy dietary patterns, and physical inactivity.^28,29^ Using one of the most recent NHANES cohorts, we found that even among food-secure individuals, the average HEI score was 52.1. This reflects a diet that “needs improvement” according to the federal guidelines, despite investment in national initiatives aimed at improving dietary habits for the general adult population, such as the Dietary Guidelines for Americans, published every 5 years since 1980, and the MyPlate initiative, which provides accessible dietary recommendations and educational resources.^30,31^ These data are consistent with the previously published studies that have demonstrated only modest improvement in diet quality in the US over the past decades with persistent or worsening disparities in diet quality by race/ethnicity, income, and education.^27,32^

Our results revealed an association between low diet quality and higher CKD prevalence among individuals at highest risk for kidney disease. Every 10-point higher diet quality score was associated with lower odds of CKD as much as 24% and 20% among individuals with DM or HTN, respectively. Several observational studies show that the findings aligned with these results. Maroto-Rodriguez et al. recently demonstrated that, among British adults, higher adherence to the alternate Mediterranean, Dietary Approaches to Stop Hypertension, and healthy plant-based eating patterns was associated with a lower risk of incident CKD, while greater adherence to unhealthy, high-inflammatory plant-based diet patterns was associated with an increased risk.^33^ Similarly, a recent meta-analysis of six studies, comprising 568,213 participants and 16,694 cases of CKD, by Mozaffari et al. found a statistically significant inverse association between adherence to Dietary Approaches to Stop Hypertension dietary patterns (examples of high-quality diets) and the risk of CKD.^34^ Our study adds to the literature by demonstrating the strong association between diet quality and CKD prevalence particularly in people at high risk for CKD, namely those with DM or HTN.

Healthy eating patterns have consistently been shown to reduce the incidence of diabetes and help improve both HTN and DM control.^35–38^ However, limited data exist regarding the impact of diet interventions on the kidney-related complications of DM and HTN. In the Look A head Trial, a randomized intervention trial comparing an intensive lifestyle intervention (combining caloric restriction, increased physical activity, and behavioral counseling) with a diabetes support and education program, the incidence rate of very high–risk CKD (defined as an eGFR <30 mL/min/1.73 m^2^ regardless of albumin-to-creatinine ratio, eGFR <45 mL/min/1.73 m^2^ and albumin-to-creatinine ratio ≥30 mg albumin/g creatinine, or an eGFR <60 mL/min/1.73 m^2^ and albumin-to-creatinine ratio >300 mg/g) was 31% lower in the intervention group over a median of 9.6 years of follow-up.^39^ Alongside these data, strong associations between diet quality and CKD prevalence demonstrated in our study underscore the need to study reasons for poor diet quality.

Food insecurity has always been considered an important barrier to consuming high-quality diets. We observed a prevalence of food insecurity among this population as high as 16%, with an additional 10% experiencing marginal food security. We found that diet quality, as reflected by HEI-2020 scores, progressively declined with worsening food security status in the unadjusted model, but the association was attenuated after adjustment for other socioeconomic factors, such as educational attainment and poverty-income ratio. These data are somewhat different from prior studies by Leung et al., focusing on older adults in previous NHANES cycles, which showed a small but statistically significant association between food insecurity and diet quality in some racial groups and older adults.^15,40^ These seemingly discrepant results might be related to our study, looking at the overall population rather than specific racial or age groups. The differences underscore that the relationship between food insecurity and diet quality is complex and warrants further investigation. It is possible that food insecurity is a better measure of food quantity rather than quality of diet.

The integration of the concept of nutritional security, which is defined as “consistent access, availability, and affordability of foods that promote health and prevent disease”, presents a promising approach to prioritizing diet quality in both public health initiatives and healthcare-based food as medicine interventions.^41^ Unlike traditional measures of food insecurity, which primarily assess food quantity and availability, nutritional security shifts the focus on access to high-quality foods that promote health and prevent disease. Our findings suggest that food insecurity may be associated with poor diet quality through complex pathways influenced by income and education and that addressing broader socioeconomic disparities may be critical in closing the diet quality gap that exits across underserved socioeconomic groups.

The primary limitation of this study is its cross-sectional design, which limits the ability to establish causal relationships. Additionally, we could not rule out the potential for unmeasured confounding factors, such as neighborhood food availability or other indicators of socioeconomic status that impact the association between diet quality and CKD prevalence. The use of a composite index as the main exposure could also contribute to attenuated results, as it may mask the effects of individual dietary components. Finally, misclassification bias (related to recall bias) and selection bias (due to exclusions or missing data) might impact the results. Despite these limitations, the study has several notable strengths. These include a large, nationally representative sample size, the use of recently collected data on food insecurity and dietary intake, and a focus on overall diet quality assessed using the most current HEI-2020.

## Conclusion

Conducted using the most recent prepandemic NHANES cohort with comprehensive data, this study highlights the continued relevance of addressing suboptimal diet quality as a critical issue affecting patients with chronic diseases, including CKD. Our findings indicate the need for further research to understand what factors in addition to food insecurity affect diet quality. The results also encourage the exploration of potential causality between diet quality and kidney health among individuals at risk for CKD. This could include prospective studies that investigate the impact of medical nutrition therapy, a Medicare-covered benefit for individuals with kidney disease that consists of a comprehensive nutrition assessment conducted by a registered dietitian, and other tailored dietary interventions in high-risk populations for preventing the development or delaying the progression of kidney disease.

## Practical Application

In an era where the tools in a box of clinicians are increasing to optimize kidney health, coupling those with interventions that promote healthy diets is essential for preventing and treating kidney disease. It is incumbent upon the kidney community to engage with the Food as Medicine initiatives around the country to develop or refine solutions to improve diet quality and further understand and address the impact of food insecurity, which are tailored to the needs of people with or at-risk kidney disease.

## Supplementary Material

Supplementary data

Supplementary Data

Supplementary data related to this article can be found at https://doi.org/10.1053/j.jrn.2025.10.004.

## Figures and Tables

**Table 1.** Demographics and Baseline Characteristics for Overall Population and for People With Diabetes and Those With Hypertension, NHANES 2017–2020

Variable	Overall	Diabetes	Hypertension

	N = 220,084,838	N = 31,440,943	N = 96,121,409

	n = 5,974	n = 1,146	n = 3,055

Age (y), mean (SD)	48.67 (17.43)	61.19 (13.12)	58.37 (15.07)
Gender, n (%)			
female	3,127 (52%)	540 (47%)	1,509 (49%)
male	2,847 (48%)	606 (53%)	1,546 (51%)
Race/ethnicity, n (%)			
White	2,208 (64%)	366 (61%)	1,172 (66%)
Black	1,619 (11%)	346 (13%)	1,003 (13%)
Hispanic	1,261 (16%)	271 (16%)	511 (12%)
Asian	600 (5.7%)	99 (5.2%)	229 (4.6%)
Other	286 (3.9%)	64 (5.7%)	140 (4.6%)
Food security status, n (%)			
Secure	3,808 (74%)	683 (72%)	1,970 (75%)
Marginal	852 (10%)	166 (10%)	413 (8.7%)
Insecure	1,314 (16%)	297 (17%)	672 (16%)
Poverty ratio, median (IQR)	3.40 (1.71–5.00)	2.96 (1.54–5.00)	3.18 (1.69–5.00)
Education level, n (%)			
College degree	1,564 (34%)	212 (24%)	706 (27%)
Some college education	2,035 (30%)	396 (32%)	1,052 (33%)
High school diploma	1,391 (26%)	287 (29%)	782 (30%)
Less than high school	939 (9.1%)	249 (15%)	510 (11%)
Body mass index (kg/m^2^), mean (SD)	28.60 (24.70, 33.30)	32.10 (28.30, 36.90)	30.20 (26.40, 35.20)
Ever smoked, n (%)	2,501 (41%)	549 (47%)	1,476 (49%)
Diabetes, n (%)	1,146 (14.3%)	1,146 (100.0%)	905 (26.0%)
Hypertension, n (%)	3,055 (45.8%)	905 (81.5%)	3,055 (100.0%)
Obesity, n (%)	2,633 (42.2%)	701 (64.5%)	1,538 (52.0%)
Heart failure, n (%)	210 (2.42%)	104 (8.70%)	182 (4.90%)
HEI 2020 Total score, mean (SD)	50.93 (12.15)	51.60 (12.23)	51.15 (11.85)

N, weighted count; n, unweighted count; HEI, healthy eating index; IQR, interquartile range; SD, standard deviation.

**Table 2.** Relation of Diet Quality Measured by HEI-2020 to CKD, NHANES 2017–2020

Variable	Overall Population		People with DM		People with HTN	
	OR* (95% CI)	*P* Value	OR (95% CI)	*P* Value	OR (95% CI)	*P* Value

Intercept	0.03 (0.01, 0.08)	<.001	0.10 (0.02, 0.53)	.01	0.03 (0.01, 0.09)	<.001
**Overall HEI score by 10 pts**	**0.88 (0.78, 1.01)**	**.06**	**0.76(0.61, 0.95)**	**.02**	**0.80 (0.69, 0.93)**	**.01**
**Age (y)**	1.03 (1.02, 1.05)	<.001	1.03 (1.01, 1.05)	.005	1.05 (1.04, 1.07)	<.001
**Male gender**	0.74 (0.54, 1.00)	.05	1.2 (0.73, 1.95)	.43	0.94 (0.68, 1.30)	.67
**Race/ethnicity**		.52		.35		.22
White	**Ref** **		**Ref**		**Ref**	
Black	1.30 (0.90, 1.90)	.15	1.49 (0.91, 2.43)	.1	1.55 (1.04, 2.31)	.03
Hispanic	1.13 (0.80, 1.59)	.46	2.29 (0.95, 5.49)	.06	1.11 (0.65, 1.90)	.67
Asian	1.07 (0.58, 1.99)	.81	1.52 (0.63, 3.68)	.31	1.24 (0.58, 2.67)	.55
Other	1.07 (0.58, 1.95)	.81	2.2 (0.78, 6.25)	.12	1.34 (0.61, 2.96)	.43
Education level		.27		.14		.14
College degree	**Ref**		**Ref**		**Ref**	
Some college education	1.16 (0.78, 1.74)	.41	1 (0.50, 2.01)	>.999	1.01 (0.61, 1.66)	.98
High school diploma	1.15 (0.72, 1.85)	.51	0.999 (0.525, 1.90)	.997	0.82 (0.55, 1.23)	.30
Less than high school	1.48 (0.95, 2.30)	.08	1.75 (0.88, 3.51)	.10	1.31 (0.85, 2.02)	.19
**Income category** †		.42		.51		.18
>2.85	**Ref**		**Ref**		**Ref**	
1.85–2.85	1.30 (0.89, 1.91)	.15	1.41 (0.72, 2.73)	.28	1.44 (0.97, 2.14)	.07
1.3–1.85	1.40 (0.83, 2.38)	.18	0.88 (0.50, 1.56)	.63	1.65 (0.94, 2.92)	.08
<1.3	1.25 (0.81, 1.91)	.27	0.93 (0.54, 1.62)	.79	1.41 (0.87, 2.31)	.15
**Obesity**	1.02 (0.79, 1.32)	.86	1.49 (0.97, 2.28)	.07	1.02 (0.77, 1.36)	.86
**Diabetes**	2.69 (2.13, 3.39)	<.001	—		2.50 (1.83, 3.41)	<.001
**Hypertension**	1.83 (1.39, 2.41)	<.001	1.96 (1.05, 3.65)	.04	—	

From logistic regression analyses adjusted for age, gender, race-ethnicity, education level, poverty income ratio category, comorbidities when applicable (DM and HTN) and obesity.

Outcomes for fully adjusted logistic regression models for the overall population, people with DM and people with HTN are shown in bold.

CI, confidence interval.

* OR: weight fully adjusted odds ratio for diet quality on CKD prevalence for every 10-point increase in HEI-2020.

** Reference category.

† Income categories are presented as percentages of the federal poverty level for a household of four, according to the US Department of Agriculture 2024–2025 guidelines, and corresponded to the following gross monthly incomes in US dollars: >2.85 = > $7,410. 1.85–2.85 = $4,810–$7,410. 1.3 –1.85 = $3,380 –$4,810. <1.3 =< $3,380.

**Table 3.** Relation of Food Security Status to Diet Quality Measured by HEI-2020, NHANES 2017–2020

Variable	Overall Population	*P* Value	People With DM	*P* Value	People With HTN	*P* Value
	Beta (95% CI)		Beta (95% CI)		Beta (95% CI)	

Intercept	49.1 (46.1, 52.1)	<0.001	42.50 (32.0, 53.0)	<0.001	46.0 (40.4, 51.6)	<0.001
Food security status		0.23		0.85		0.56
Secure	**Ref**		**Ref**		**Ref**	
Marginal	−0.79 (−3.06, 1.49)	0.45	0.28 (−3.58, 4.15)	0.87	0.56 (−2.01, 3.13)	0.63
Insecure	−1.44 (−3.35, 0.46)	0.12	0.85 (−2.51, 4.21)	0.58	−0.66 (−2.70, 1.39)	0.49
Age (y)	0.15 (0.11, 0.19)	<0.001	0.17 (0.07, 0.28)	0.01	0.16 (0.10, 0.23)	<0.001
Male gender	−1.82 (−3.01, −0.63)	0.01	−1.43 (−4.14, 1.28)	0.26	−0.83 (−2.37, 0.71)	0.25
Race/ethnicity		<0.001		0.002		0.002
White	**Ref**		**Ref**		**Ref**	
Black	1.09 (−0.53, 2.71)	0.16	1.99 (−1.71,5.69)	0.26	1.52 (−0.62, 3.66)	0.14
Hispanic	3.35 (1.43, 5.26)	0.004	3.97 (0.61, 7.32)	0.03	3 (0.64, 5.36)	0.02
Asian	5.28 (3.82, 6.73)	<0.001	9.45 (5.52, 13.40)	<0.001	7.06 (4.50, 9.63)	<0.001
Other	0.07 (−2.65, 2.79)	0.95	−0.16 (−6.10, 5.80)	0.95	1.23 (−2.76, 5.22)	0.50
Education level		0.004		0.69		0.01
College degree	**Ref**		**Ref**		**Ref**	
Some college education	−4.43 (−6.52,-2.34)	0.001	−1.99 (−7.08, 3.10)	0.4	−3.48 (−5.97, −1.00)	0.01
High school diploma	−5.46 (−7.82, −3.09)	<0.001	−0.77 (−4.95, 3.40)	0.69	−4.91 (−7.08, −2.73)	<0.001
Less than high school	−4.30 (−7.29, −1.31)	0.01	−2.03 (−7.16, 3.11)	0.40	−3.15 (−6.32, 0.02)	0.05
Income category		0.10		0.15		0.09
>2.85	**Ref**		**Ref**		**Ref**	
1.85–2.85	−2.50 (−4.86, −0.15)	0.04	−4.11 (−8.74, 0.52)	0.08	−2.33 (−4.75, 0.09)	0.06
1.3–1.85	−1.88 (−3.92, 0.17)	0.07	−1.14 (−5.01,2.73)	0.52	−1.57 (−4.26, 1.12)	0.22
<1.3	−2.00 (−3.71, −0.28)	0.03	−3.49 (−7.29, 0.30)	0.07	−2.97 (−5.45, −0.49)	0.02
Obesity	−2.06 (−3.24, −0.88)	0.004	0.12 (−1.54, 1.77)	0.88	−1.55 (−3.13, 0.04)	0.05
Diabetes status	0.45 (−1.54, 2.43)	0.62	—	—	0.67 (−1.86, 3.20)	0.56
Hypertension	−0.73 (−2.44, 0.97)	0.35	0.68 (−4.47, 5.84)	0.77	—	—

From linear regression analyses adjusted for age, gender, race-ethnicity, education level, poverty income ratio category, comorbidities when applicable (DM and HTN) and obesity.

CI, confidence Interval; DM, diabetes mellitus; HTN, hypertension; Ref, reference Category.

## References

[1] Shan Z, Li Y, Baden MY, Association between healthy eating patterns and risk of cardiovascular disease. JAMA Intern Med. 2020;180:1090–1100. 10.1001/jamainternmed.2020.2176 PMC7296454 32539102

[2] Shan Z, Wang F, Li Y, Healthy eating patterns and risk of total and cause-specific mortality. JAMA Intern Med. 2023;183:142–153. 10.1001/jamainternmed.2022.6117 PMC9857813 36622660

[3] Hu EA, Coresh J, Anderson CAM, Adherence to healthy dietary patterns and risk of CKD progression and all-cause mortality: findings from the CRIC (Chronic Renal Insufficiency Cohort) study. Am J Kidney Dis. 2021;77:235–244. 10.1053/j.ajkd.2020.04.019 PMC7855760 32768632

[4] Schwingshackl L, Hoffmann G. Diet quality as assessed by the healthy eating index, the alternate healthy eating index, the dietary approaches to stop hypertension score, and health outcomes: a systematic review and meta-analysis of cohort studies. J Acad Nutr Diet. 2015;115:780–800.e5. 10.1016/j.jand.2014.12.009 25680825

[5] Mozaffarian D. Dietary and policy priorities for cardiovascular disease, diabetes, and obesity: a comprehensive review. Circulation. 2016;133:187–225. 10.1161/CIRCULATIONAHA.115.018585 PMC4814348 26746178

[6] Gao Y, Yang A, Zurbau A, Gucciardi E. The effect of food is medicine interventions on diabetes-related health outcomes among low-income and food-insecure individuals: a systematic review and meta-analysis. Can J Diabetes. 2023;47:143–152. 10.1016/j.jcjd.2022.11.001 36470724

[7] Filippou CD, Tsioufis CP, Thomopoulos CG, Dietary Approaches to Stop Hypertension (DASH) diet and blood pressure reduction in adults with and without hypertension: a systematic review and meta-analysis of randomized controlled trials. Adv Nutr Bethesda Md. 2020;11:1150–1160. 10.1093/advances/nmaa041 PMC7490167 32330233

[8] Food is medicine: A project to unify and advance collective action | odphp.health.gov. https://odphp.health.gov/our-work/nutrition-physical-activity/food-medicine. Accessed March 9, 2025.

[9] Mozaffarian D, Blanck HM, Garfield KM, Wassung A, Petersen R. A food is medicine approach to achieve nutrition security and improve health. Nat Med. 2022;28:2238–2240. 10.1038/s41591-022-02027-3 PMC10851911 36202998

[10] Food is medicine initiative - food & society. https://aspenfood.org/fim/. Accessed February 19, 2025.

[11] USDA releases study on hurdles to healthy eating on SNAP | home. https://www.usda.gov/about-usda/news/press-releases/2021/06/23/usda-releases-study-hurdles-healthy-eating-snap. Accessed April 3, 2025.

[12] USDA ERS - food security in the U.S. https://www.ers.usda.gov/topics/food-nutrition-assistance/food-security-in-the-u-s/. Accessed August 15, 2022.

[13] Usda ERS - definitions of food security. https://www.ers.usda.gov/topics/food-nutrition-assistance/food-security-in-the-u-s/definitions-of-food-security/. Accessed April 6, 2024.

[14] Arenas DJ, Zhou S, Thomas A, Wang J, Arroyo GV, Bash K. Negative Health Outcomes Associated With Food Insecurity Status in the United States of America: a Systematic Review of Peer-Reviewed Studies. Charlottesville, VA: Center for Open Science; 2018 10.31232/osf.io/uk9xw. OSF Preprints.

[15] Leung CW, Tester JM. The association between food insecurity and diet quality varies by race/ethnicity: an analysis of national health and nutrition examination survey 2011–2014 results. J Acad Nutr Diet. 2019;119:1676–1686. 10.1016/j.jand.2018.10.011 PMC6584552 30579633

[16] Stierman B, Afful J, Carroll MD, National health and nutrition examination survey 2017-March 2020 prepandemic data files-development of files and prevalence estimates for selected health outcomes. Natl Health Stat Rep. 2021;158 10.15620/cdc:106273. PMC11513744 39380201

[17] Zhan JJ, Hodge RA, Dunlop AL, Dietaryindex: a user-friendly and versatile R package for standardizing dietary pattern analysis in epidemiological and clinical studies. bioRxiv. 2023; 10.1101/2023.08.07.548466. PMC11600030 39182618

[18] Xu Z Improving the dietaryindex R package: a proposal to include additional components for more accurate DII computation in NHANES. Am J Clin Nutr. 2025;121:174–175. 10.1016/j.ajcnut.2024.10.023 39528051

[19] Healthy Eating Index (HEI) | food and nutrition service. https://www.fns.usda.gov/cnpp/healthy-eating-index-hei. Accessed December 29, 2024.

[20] Shams-White MM, Pannucci TE, Lerman JL, Healthy eating Index-2020: review and update process to reflect the dietary guidelines for Americans,2020–2025. J Acad Nutr Diet. 2023;123:1280–1288. 10.1016/j.jand.2023.05.015 PMC10524328 37201748

[21] USDA ERS - survey tools. https://www.ers.usda.gov/topics/food-nutrition-assistance/food-security-in-the-u-s/survey-tools/. Accessed June 3, 2024.

[22] Bureau UC. Poverty in the United States: 2023. Census.gov. https://www.census.gov/library/publications/2024/demo/p60-283.html. Accessed November 19, 2024.

[23] Child nutrition programs income eligibility guidelines (2021–22). Food Nutr Serv. https://www.fns.usda.gov/cn/fr-030421. Accessed March 9, 2025.

[24] Inker LA, Eneanya ND, Coresh J, New Creatinine- and cystatin C–Based equations to estimate GFR without race. N Engl J Med. 2021;385:1737–1749. 10.1056/NEJMoa2102953 PMC8822996 34554658

[25] R: The R project for statistical computing. https://www.r-project.org/. Accessed March 9, 2025.

[26] Lumley T, Gao P, Schneider B. Survey: analysis of complex survey samples. https://cran.r-project.org/web/packages/survey/index.html. Accessed March 9, 2025.

[27] Liu J, Mozaffarian D. Trends in diet quality among U.S. adults from 1999 to 2020 by race, ethnicity, and socioeconomic disadvantage. Ann Intern Med. 2024;177:841–850. 10.7326/M24-0190 38885507

[28] Mokdad AH, Marks JS, Stroup DF, Gerberding JL. Actual causes of death in the United States, 2000. JAMA. 2004;291:1238–1245. 10.1001/jama.291.10.1238 15010446

[29] Bauer UE, Briss PA, Goodman RA, Bowman BA. Prevention of chronic disease in the 21st century: elimination of the leading preventable causes of premature death and disability in the USA. Lancet Lond Engl. 2014;384:45–52. 10.1016/S0140-6736(14)60648-6 24996589

[30] Home | dietary guidelines for Americans. https://www.dietaryguidelines.gov/. Accessed March 20, 2025.

[31] MyPlate.gov | U.S. Department of Agriculture. https://www.myplate.gov/. Accessed March 20, 2025.

[32] Liu J, Rehm CD, Onopa J, Mozaffarian D. Trends in diet quality among youth in the United States, 1999–2016. JAMA. 2020;323:1161–1174. 10.1001/jama.2020.0878 PMC7093765 32207798

[33] Maroto-Rodriguez J, Ortolá R, Cabanas-Sanchez V, Martinez-Gomez D, Rodriguez-Artalejo F, Sotos-Prieto M. Diet quality patterns and chronic kidney disease incidence: a UK biobank cohort study. Am J Clin Nutr. 2025;121:445–453. 10.1016/j.ajcnut.2024.12.005 39667719

[34] Mozaffari H, Ajabshir S, Alizadeh S. Dietary approaches to stop hypertension and risk of chronic kidney disease: a systematic review and meta-analysis of observational studies. Clin Nutr Edinb Scotl. 2020;39:2035–2044. 10.1016/j.clnu.2019.10.004 31669002

[35] Morze J, Danielewicz A, Hoffmann G, Schwingshackl L. Diet quality as assessed by the healthy eating index, alternate healthy eating index, dietary approaches to stop hypertension score, and health outcomes: a second update of a systematic review and meta-analysis of cohort studies. J Acad Nutr Diet. 2020;120:1998–2031.e15. 10.1016/j.jand.2020.08.076 33067162

[36] Quan X, Shen X, Li C, Li Y, Li T, Chen B. Adherence to the dietary approaches to stop hypertension diet reduces the risk of diabetes mellitus: a systematic review and dose-response meta-analysis. Endocrine. 2024;86:85–100. 10.1007/s12020-024-03882-5 PMC11445359 38816664

[37] Knowler WC, Barrett-Connor E, Fowler SE, Reduction in the incidence of type 2 diabetes with lifestyle intervention or metformin. N Engl J Med. 2002;346:393–403. 10.1056/NEJMoa012512 PMC1370926 11832527

[38] Theodoridis X, Chourdakis M, Chrysoula L, Adherence to the DASH diet and risk of hypertension: a systematic review and meta-analysis. Nutrients. 2023;15:3261. 10.3390/nu15143261 PMC10383418 37513679

[39] Knowler WC, Bahnson JL, Bantle JP, Effect of a long-term behavioral weight loss intervention on nephropathy in overweight or Obese adults with type 2 diabetes: the look AHEAD randomized clinical trial. Lancet Diabetes Endocrinol. 2014;2:801–809. 10.1016/S2213-8587(14)70156-1 PMC4443484 25127483

[40] Leung CW, Wolfson JA. Food insecurity among older adults: 10-year national trends and associations with diet quality. J Am Geriatr Soc. 2021;69:964–971. 10.1111/jgs.16971 PMC8341441 33403662

[41] Mozaffarian D, Fleischhacker S, Andrés JR. Prioritizing nutrition security in the US. JAMA. 2021;325:1605–1606. 10.1001/jama.2021.1915 33792612

