# Supplementary material for: Diet Quality, Food Insecurity, and Chronic Kidney Disease: Insights From the US National Health and Nutrition Examination Survey
Source: J Ren Nutr. Author manuscript; Available in PMC 2026 Oct 1. (PMC13629284; doi:10.1053/j.jrn.2025.10.004)
Supplement: Supplementary file 1 [file NIHMS2209020-supplement-Supplementary_data.zip › 1-s2.0-S1051227625002225-mmc1.docx]

Supplemental Table-1: Weighted Logistic Regression Results for HEI-2020 (per 10 pts) and CKD Across Models in People with DM, NHANES 2017-2020

|  | Model 1: Unadjusted | | Model 2: + Sociodemographics | | Model 3: + All Covariates | |
| --- | --- | --- | --- | --- | --- | --- |
| Variable | **OR^*^ (95% CI^**^)** | **p-value** | **OR (95% CI)** | **p-value** | **OR (95% CI)** | **p-value** |
| Intercept | 1.44(0.59, 3.51) | 0.41 | 0.20(0.04, 0.93) | 0.041 | 0.10 (0.02, 0.53) | 0.01 |
| Overall HEI Score by 10 pts | 0.85(0.71, 1.01) | 0.07 | 0.79(0.64, 0.97) | 0.03 | 0.76(0.61, 0.95) | 0.02 |
| **Age (years)** |  |  | 1.03(1.01, 1.06) | 0.01 | 1.03(1.01, 1.05) | 0.01 |
| **Male gender** |  |  | 1.12(0.69, 1.82) | 0.63 | 1.2(0.73, 1.95) | 0.43 |
| **Race/ethnicity** |  |  |  | 0.52 |  | 0.35 |
| White |  |  | **Ref**^†^ |  | **Ref** |  |
| Black |  |  | 1.4(0.88, 2.23) | 0.14 | 1.49(0.91, 2.43) | 0.1 |
| Hispanic |  |  | 1.79(0.79, 4.06) | 0.15 | 2.29(0.95, 5.49) | 0.06 |
| Asian |  |  | 1.21(0.53, 2.79) | 0.63 | 1.52(0.63, 3.68) | 0.31 |
| Other |  |  | 1.86(0.75, 4.61) | 0.16 | 2.2(0.78, 6.25) | 0.12 |
| **Education Level** |  |  |  | 0.12 |  | 0.14 |
| College degree |  |  | **Ref** |  | **Ref** |  |
| Some college education |  |  | 1.08(0.53, 2.18) | 0.82 | 1(0.50, 2.01) | 1 |
| High school diploma |  |  | 1.14(0.54, 2.39) | 0.71 | 0.999(0.53, 1.90) | 1 |
| Less than high school |  |  | 1.82(0.95, 3.51) | 0.07 | 1.75(0.88, 3.51) | 0.10 |
| **Poverty categor**y |  |  |  | 0.53 |  | 0.51 |
| >2.85 |  |  | **Ref** |  | **Ref** |  |
| 1.85-2.85 |  |  | 1.4(0.74, 2.63) | 0.27 | 1.41(0.72, 2.73) | 0.28 |
| 1.3-1.85 |  |  | 0.89(0.54, 1.48) | 0.63 | 0.881(0.50, 1.56) | 0.63 |
| < 1.3 |  |  | 0.91(0.55, 1.52) | 0.71 | 0.933(0.54, 1.62) | 0.79 |
| **Obesity** |  |  |  |  | 1.49(0.97, 2.28) | 0.07 |
| **Hypertension** |  |  |  |  | 1.96(1.05, 3.65) | 0.04 |

*Models adjusted for age, gender, race-ethnicity, education level, poverty income ratio category, co-morbidities when applicable (DM and HTN) and obesity.*

Abbreviations: ^*^ Odds Ratio, ^**^ Confidence interval, ^†^Reference category, HEI Healthy Eating Index
