# Supplementary material for: Diet Quality, Food Insecurity, and Chronic Kidney Disease: Insights From the US National Health and Nutrition Examination Survey
Source: J Ren Nutr. Author manuscript; Available in PMC 2026 Oct 1. (PMC13629284; doi:10.1053/j.jrn.2025.10.004)
Supplement: Supplementary file 1 [file NIHMS2209020-supplement-Supplementary_data.zip › 1-s2.0-S1051227625002225-mmc3.docx]

Supplemental Table-3: Weighted Linear Regression Results for Food Security and Diet Quality (HEI-2020) Across Models for Overall Population NHANES 2017-2020

| Variable | **Model 1: Unadjusted** | | **Model 2: + Sociodemographics** | | **Model 3: + All Covariates** | |
| --- | --- | --- | --- | --- | --- | --- |
|  | **Beta (95% CI^*^)** | **p-value** | **Beta (95% CI)** | **p-value** | **Beta (95% CI)** | **p-value** |
| **Intercept** | 52.1(51.1, 53.1) | <0.001 | 48.7(45.9, 51.4) | <0.001 | 49.1(46.1, 52.1) | <0.001 |
| **Food Security Status** |  | <0.001 |  | 0.15 |  | 0.23 |
| Secure | **Ref^**^** |  | **Ref** |  | **Ref** |  |
| Marginal | -3.83(-5.58, -2.07) | <0.001 | -1.01 (-3.18, 1.15) | 0.33 | -0.79(-3.06, 1.49) | 0.45 |
| Insecure | -4.53(-5.73, -3.33) | <0.001 | -1.68 (-3.46, 0.10) | 0.06 | -1.44(-3.35, 0.46) | 0.12 |
| **Age (years)** |  |  | 0.141(0.10, 0.18) | <0.001 | 0.151(0.108, 0.194) | <0.001 |
| **Male gender** |  |  | -1.69 (-2.70, -0.68) | 0.004 | -1.82 (-3.01, -0.63) | 0.01 |
| **Race/ethnicity** |  |  |  | <0.001 |  | <0.001 |
| White |  |  | **Ref** |  | **Ref** |  |
| Black |  |  | 0.66(-0.79, 2.11) | 0.34 | 1.09(-0.53, 2.71) | 0.16 |
| Hispanic |  |  | 3.04(1.16, 4.92) | 0.004 | 3.35(1.43, 5.26) | 0.004 |
| Asian |  |  | 5.44(4.00, 6.87) | <0.001 | 5.28(3.82, 6.73) | <0.001 |
| Other |  |  | -0.38(-2.93, 2.16) | 0.75 | 0.07 (-2.65, 2.79) | 0.95 |
| **Education level** |  |  |  | <0.001 |  | 0.004 |
| College degree |  |  | **Ref** |  | **Ref** |  |
| Some college education |  |  | -4.83 (-6.72, -2.95) | <0.001 | -4.43(-6.52, -2.34) | 0.001 |
| High school diploma |  |  | -5.87 (-8.09, -3.65) | <0.001 | -5.46(-7.82, -3.09) | <0.001 |
| Less than high school |  |  | -4.49 (-7.07, -1.91) | 0.003 | -4.30 (-7.29, -1.31) | 0.01 |
| **Income Category** |  |  |  | 0.08 |  | 0.10 |
| >2.85 |  |  | **Ref** |  | **Ref** |  |
| 1.85-2.85 |  |  | -2.15 (-4.22, -0.08) | 0.04 | -2.50(-4.86, -0.147) | 0.04 |
| 1.3-1.85 |  |  | -1.69 (-3.37, -0.02) | 0.05 | -1.88(-3.92, 0.17) | 0.07 |
| < 1.3 |  |  | -1.83(-3.38, -0.283) | 0.03 | -2.00(-3.71, -0.28) | 0.03 |
| **Obesity** |  |  |  |  | -2.06(-3.24, -0.88) | 0.004 |
| **Diabetes status** |  |  |  |  | 0.45(-1.54, 2.43) | 0.62 |
| **Hypertension** |  |  |  |  | -0.73(-2.44, 0.97) | 0.35 |

Models adjusted for age, gender, race-ethnicity, education level, poverty income ratio category, co-morbidities when applicable (DM and HTN) and obesity.

Abbreviations: * Confidence interval, ** Reference category
