# Supplementary material for: Diet Quality, Food Insecurity, and Chronic Kidney Disease: Insights From the US National Health and Nutrition Examination Survey
Source: J Ren Nutr. Author manuscript; Available in PMC 2026 Oct 1. (PMC13629284; doi:10.1053/j.jrn.2025.10.004)
Supplement: Supplementary file 1 [file NIHMS2209020-supplement-Supplementary_data.zip › 1-s2.0-S1051227625002225-mmc5.docx]

Supplemental Table-5: Weighted Linear Regression Results for Food Security and Diet Quality (HEI-2020) Across Models for People with Hypertension, NHANES 2017-2020

| Variable | **Model 1: Unadjusted** | | **Model 2: + Sociodemographics** | | **Model 3: + All Covariates** | |
| --- | --- | --- | --- | --- | --- | --- |
|  | **Beta (95% CI^*^)** | **p-value** | **Beta (95% CI)** | **p-value** | **Beta (95% CI)** | **p-value** |
| **Intercept** | 52 (50.7, 53.2) | <0.001 | 44.6 (40.3, 49.0) | <0.001 | 46 (40.4, 51.6) | <0.001 |
| **Food Security Status** |  | <0.001 |  | 0.47 |  | 0.56 |
| Secure | **Ref^**^** |  | **Ref** |  | **Ref** |  |
| Marginal | -1.77 (-4.20, 0.651) | 0.144 | 0.55 (-1.94, 3.04) | 0.64 | 0.56 (-2.01, 3.13) | 0.63 |
| Insecure | -3.93 (-5.37, -2.49) | <0.001 | -0.80 (-2.76, 1.17) | 0.39 | -0.66 (-2.70, 1.39) | 0.49 |
| **Age (years)** |  |  | 0.17 (0.11, 0.24) | <0.001 | 0.16 (0.09, 0.23) | <0.001 |
| **Male gender** |  |  | -0.74 (-2.13, 0.66) | 0.27 | -0.83 (-2.37, 0.71) | 0.25 |
| **Race/ethnicity** |  |  |  | <0.001 |  | 0.002 |
| White |  |  | **Ref** |  | **Ref** |  |
| Black |  |  | 1.42 (-0.62, 3.46) | 0.15 | 1.52 (-0.62, 3.66) | 0.14 |
| Hispanic |  |  | 2.95 (0.63, 5.28) | 0.018 | 3 (0.64, 5.36) | 0.018 |
| Asian |  |  | 7.51 (5.14, 9.87) | <0.001 | 7.06 (4.50, 9.63) | <0.001 |
| Other |  |  | 1.16 (-2.67, 4.99) | 0.52 | 1.23 (-2.76, 5.22) | 0.50 |
| **Education level** |  |  |  | 0.002 |  | 0.005 |
| College degree |  |  | **Ref** |  | **Ref** |  |
| Some college education |  |  | -3.57 (-6.01, -1.13) | 0.008 | -3.48 (-5.97, -1.00) | 0.011 |
| High school diploma |  |  | -5.07 (-7.19, -2.95) | <0.001 | -4.91 (-7.08, -2.73) | <0.001 |
| Less than high school |  |  | -3.03 (-6.00, -0.056) | 0.05 | -3.15 (-6.32, 0.02) | 0.05 |
| **Income Category** |  |  |  | 0.11 |  | 0.09 |
| >2.85 |  |  | **Ref** |  | **Ref** |  |
| 1.85-2.85 |  |  | -2.14 (-4.42, 0.130) | 0.06 | -2.33 (-4.75, 0.09) | 0.06 |
| 1.3-1.85 |  |  | -1.59 (-4.17, 0.99) | 0.20 | -1.57 (-4.26, 1.12) | 0.22 |
| < 1.3 |  |  | -2.71 (-5.10, -0.32) | 0.03 | -2.97 (-5.45, -0.49) | 0.02 |
| **Obesity** |  |  |  |  | -1.55 (-3.13, 0.04) | 0.05 |
| **Diabetes** |  |  |  |  | 0.67 (-1.86, 3.20) | 0.56 |

Models adjusted for age, gender, race-ethnicity, education level, poverty income ratio category, co-morbidities when applicable (DM and HTN) and obesity.

Abbreviations: * CI: Confidence interval, ** Ref: Reference Category
