# Supplementary material for: Diet Quality, Food Insecurity, and Chronic Kidney Disease: Insights From the US National Health and Nutrition Examination Survey
Source: J Ren Nutr. Author manuscript; Available in PMC 2026 Oct 1. (PMC13629284; doi:10.1053/j.jrn.2025.10.004)
Supplement: Supplementary file 1 [file NIHMS2209020-supplement-Supplementary_data.zip › 1-s2.0-S1051227625002225-mmc4.docx]

Supplemental Table-4: Weighted Linear Regression Results for Food Security and Diet Quality (HEI-2020) Across Models for People with Diabetes

| Variable | **Model 1: Unadjusted** | | **Model 2: + Sociodemographics** | | **Model 3: + All Covariates** | |
| --- | --- | --- | --- | --- | --- | --- |
|  | **Beta (95% CI^*^)** | **p-value** | **Beta (95% CI)** | **p-value** | **Beta (95% CI)** | **p-value** |
| **Intercept** | 52.0(49.60, 54.50) | <0.001 | 43.1(33.5, 52.7) | <0.001 | 42.5(32.0, 53.0) | <0.001 |
| **Food Security Status** |  | 0.336 |  | 0.962 |  | 0.85 |
| Secure | **Ref^**^** |  | **Ref** |  | **Ref** |  |
| Marginal | -1.35(-4.49, 1.80) | 0.384 | 0.120 (-3.59, 3.83) | 0.945 | 0.28(-3.58, 4.15) | 0.87 |
| Insecure | -1.64(-3.92, 0.63) | 0.148 | 0.380(-2.68, 3.44) | 0.79 | 0.85(-2.51, 4.21) | 0.58 |
| **Age (years)** |  |  | 0.170(0.070, 0.269) | 0.003 | 0.17(0.07, 0.28) | 0.005 |
| **Male gender** |  |  | -1.25 (-3.76, 1.25) | 0.294 | -1.43 (-4.14, 1.28) | 0.26 |
| **Race/ethnicity** |  |  |  | 0.001 |  | 0.002 |
| White |  |  | **Ref** |  | **Ref** |  |
| Black |  |  | 1.71(-1.60, 5.02) | 0.279 | 1.99(-1.71, 5.69) | 0.26 |
| Hispanic |  |  | 2.95 (0.625, 5.28) | 0.071 | 3.97(0.61, 7.32) | 0.03 |
| Asian |  |  | 7.51 (5.14, 9.87) | <0.001 | 9.45(5.52, 13.4) | <0.001 |
| Other |  |  | 1.16 (-2.67, 4.99) | 0.873 | -0.16(-6.10, 5.80) | 0.95 |
| **Education level** |  |  |  | 0.811 |  | 0.69 |
| College degree |  |  | **Ref** |  | **Ref** |  |
| Some college education |  |  | -1.67(-6.61, 3.27) | 0.473 | -1.99(-7.08, 3.10) | 0.4 |
| High school diploma |  |  | -0.483(-4.60, 3.63) | 0.801 | -0.77(-4.95, 3.40) | 0.69 |
| Less than high school |  |  | -1.23 (-6.07, 3.61) | 0.587 | -2.03(-7.16, 3.11) | 0.40 |
| **Income Category** |  |  |  | 0.137 |  | 0.15 |
| >2.85 |  |  | **Ref** |  | **Ref** |  |
| 1.85-2.85 |  |  | -3.83 (-8.14, 0.473) | 0.076 | -4.11(-8.74, 0.516) | 0.08 |
| 1.3-1.85 |  |  | -1.21(-4.87, 2.44) | 0.48 | -1.14 (-5.01, 2.73) | 0.52 |
| < 1.3 |  |  | -3.37 (-6.80, 0.061) | 0.054 | -3.49(-7.29, 0.300) | 0.07 |
| **Obesity** |  |  |  |  | 0.115 (-1.54, 1.77) | 0.88 |
| **Hypertension** |  |  |  |  | 0.681 (-4.47, 5.84) | 0.77 |

Models adjusted for age, gender, race-ethnicity, education level, poverty income ratio category, co-morbidities when applicable (DM and HTN) and obesity.

Abbreviations: *Confidence interval, ** Reference Category
