# Supplementary material for: Diet Quality, Food Insecurity, and Chronic Kidney Disease: Insights From the US National Health and Nutrition Examination Survey
Source: J Ren Nutr. Author manuscript; Available in PMC 2026 Oct 1. (PMC13629284; doi:10.1053/j.jrn.2025.10.004)
Supplement: Supplementary file 1 [file NIHMS2209020-supplement-Supplementary_data.zip › 1-s2.0-S1051227625002225-mmc2.docx]

Supplemental Table-2: Weighted Logistic Regression Results for Diet Quality as measured by HEI-2020 (per 10 pts) and CKD Across Models in People with Hypertension, NHANES 2017-2020

|  | **Model 1: Unadjusted** | | **Model 2: + Sociodemographics** | | **Model 3: + All Covariates** | |
| --- | --- | --- | --- | --- | --- | --- |
| **Variable** | **OR^*^ (95% CI^**^)** | **p-value** | **OR (95% CI)** | **p-value** | **OR (95% CI)** | **p-value** |
| **Intercept** | 0.50(0.30, 0.84) | 0.01 | 0.03(0.01, 0.08) | <0.001 | 0.03(0.01, 0.09) | <0.001 |
| **Overall HEI Score by 10 pts** | 0.92(0.84, 1.01) | 0.081 | 0.81(0.71, 0.93) | 0.01 | 0.80 (0.69, 0.93) | 0.01 |
| **Age (years)** |  |  | 1.06(1.04, 1.07) | <0.001 | 1.05(1.04,1.07) | <0.001 |
| **Male gender** |  |  | 0.99(0.75,1.32) | 0.96 | 0.94(0.68, 1.30) | 0.67 |
| **Race/ethnicity** |  |  |  | 0.21 |  | 0.22 |
| White |  |  | **Ref** |  | **Ref** |  |
| Black |  |  | 1.56(1.07, 2.28) | 0.02 | 1.55(1.04, 2.31) | 0.03 |
| Hispanic |  |  | 1.27(0.77, 2.11) | 0.32 | 1.11(0.65,1.90) | 0.67 |
| Asian |  |  | 1.32(0.63, 2.76) | 0.43 | 1.24(0.58,2.67) | 0.55 |
| Other |  |  | 1.45(0.72, 2.90) | 0.27 | 1.34(0.61,2.96) | 0.43 |
| **Education Level** |  |  |  | 0.10 |  | 0.14 |
| College degree |  |  | **Ref**^†^ |  | **Ref** |  |
| Some college education |  |  | 1.06(0.64,1.78) | 0.80 | 1.01(0.61, 1.66) | 0.98 |
| High school diploma |  |  | 0.87(0.57, 1.32) | 0.47 | 0.82(0.55, 1.23) | 0.30 |
| Less than high school |  |  | 1.39(0.89, 2.16) | 0.13 | 1.31(0.85, 2.02) | 0.19 |
| **Poverty category** |  |  |  | 0.14 |  | 0.18 |
| >2.85 |  |  | **Ref** |  | **Ref** |  |
| 1.85-2.85 |  |  | 1.48(0.98, 2.23) | 0.06 | 1.44(0.97, 2.14) | 0.07 |
| 1.3-1.85 |  |  | 1.75(1.05, 2.91) | 0.03 | 1.65(0.94, 2.92) | 0.08 |
| < 1.3 |  |  | 1.50(0.93, 2.41) | 0.09 | 1.41(0.87, 2.31) | 0.15 |
| **Obesity** |  |  |  |  | 1.02(0.77,1.36) | 0.86 |
| **Diabetes** |  |  |  |  | 2.50(1.83, 3.41) | <0.001 |

*Models adjusted for age, gender, race-ethnicity, education level, poverty income ratio category, co-morbidities when applicable (DM and HTN) and obesity.*

Abbreviations: ^*^ Odds Ratio, ^**^ Confidence interval, ^†^Reference category, HEI Healthy Eating Index
