# Supplementary material for: Diet Quality, Food Insecurity, and Chronic Kidney Disease: Insights From the US National Health and Nutrition Examination Survey
Source: J Ren Nutr. Author manuscript; Available in PMC 2026 Oct 1. (PMC13629284; doi:10.1053/j.jrn.2025.10.004)
Supplement: Supplementary file 1 [file NIHMS2209020-supplement-Supplementary_data.zip › 1-s2.0-S1051227625002225-mmc6.docx]

Supplemental Table-6: Healthy Eating Index Scores and Component Scores for the Overall Population, Participants with Diabetes, and Participants with Hypertension — NHANES 2017–2020

| Variable | Overall | Diabetes | Hypertension |
| --- | --- | --- | --- |
|  | **N = 220,084,838***^1^* | **N = 31,440,943***^1^* | **N = 96,121,409***^1^* |
|  | n=5,974^2^ | n=1,146 | n=3,055 |
| **HEI 2020 Total Score, mean (SD)** | 50.93 (12.15) | 51.60 (12.23) | 51.15 (11.85) |
| **Total Fruit** | 1.85 (0.11 - 3.37) | 2.50 (0.23 - 3.98) | 2.05 (0.18 - 3.59) |
| **Whole Fruit** | 2.34 (0.00 - 3.77) | 2.50 (0.12 - 5.00) | 2.50 (0.00 - 4.00) |
| **Total Vegetables** | 3.03 (1.95 - 4.13) | 3.08 (2.12 - 4.17) | 3.07 (1.98 - 4.17) |
| **Greens and Beans** | 1.04 (0.00 - 2.50) | 1.29 (0.00 - 2.50) | 0.78 (0.00 - 2.50) |
| **Total Protein Foods** | 4.75 (3.80 - 5.00) | 4.94 (3.91 - 5.00) | 4.71 (3.83 - 5.00) |
| **Seafood and Plant Protein** | 2.50 (0.45 - 4.12) | 2.50 (0.27 - 4.16) | 2.50 (0.29 - 4.12) |
| **Whole Grains** | 1.57 (0.00 - 4.29) | 1.87 (0.00 - 4.76) | 1.62 (0.00 - 4.46) |
| **Dairy** | 4.74 (2.55 - 6.68) | 4.58 (2.39 - 6.32) | 4.68 (2.40 - 6.65) |
| **Fatty Acids Ratio** | 5.00 (2.67 - 7.03) | 5.00 (2.83 - 6.97) | 5.00 (2.46 - 7.05) |
| **Refined Grains** | 6.66 (4.41 - 8.93) | 6.81 (4.49 - 8.99) | 7.17 (4.73 - 9.12) |
| **Sodium** | 4.64 (2.41 - 6.63) | 4.21 (1.65 - 6.56) | 4.71 (2.42 - 6.81) |
| **Added Sugars** | 7.99 (5.38 - 9.66) | 8.42 (6.41 - 9.89) | 7.99 (5.62 - 9.53) |
| **Saturated Fats** | 5.20 (3.20 - 7.59) | 4.99 (2.50 - 7.39) | 5.11 (2.94 - 7.45) |
| ^1^ N: weighted count, ^2^n: unweighted count, Mean (SD; Standard Deviation); Median (Q1 - Q3); HEI: Healthy eating index | | | |

Components scored 0–5: Total Fruits, Whole Fruits, Total Vegetables, Greens and Beans, Total Protein Foods, and Seafood and Plant Proteins. Components scored 0–10: Whole Grains, Dairy, Fatty Acids, Refined Grains, *Sodium, *Added Sugars, and *Saturated Fats (*=Reverse scored). The total HEI score is the sum of all component scores.
